# Intracranial Non-traumatic Aneurysms in Children and Adolescents

**DOI:** 10.2174/221155281120100005

**Published:** 2013-11

**Authors:** Angelika Sorteberg, Daniel Dahlberg

**Affiliations:** Dept of Neurosurgery, Oslo University Hospital – Rikshsospitalet, The National Hospital, Oslo, Norway

**Keywords:** Cerebral vasospasm, giant aneurysm, intracranial aneurysm, outcome, pediatric, subarachnoid hemorrhage.

## Abstract

An intracranial aneurysm in a child or adolescent is a rare, but potentially devastating condition. As little as approximately 1200 cases are reported between 1939 and 2011, with many of the reports presenting diverting results. There is consensus, though, in that pediatric aneurysms represent a pathophysiological entity different from their adult counterparts. In children, there is a male predominance. About two-thirds of pediatric intracranial aneurysms become symptomatic with hemorrhage and the rate of re-hemorrhage is higher than in adults. The rate of hemorrhage from an intracranial aneurysm peaks in girls around menarche. The most common aneurysm site in children is the internal carotid artery, in particular at its terminal ending. Aneurysms in the posterior circulation are more common in children than adults. Children more often develop giant aneurysms, and may become symptomatic from the mass effect of the aneurysm (tumorlike symptoms). The more complex nature of pediatric aneurysms poses a larger challenge to treatment alongside with higher demands to the durability of treatment. Outcome and mortality are similar in children and adults, but long-term outcome in the pediatric population is influenced by the high rate of aneurysm recurrences and de novo formation of intracranial aneurysms. This urges the need for life-long follow-up and screening protocols.

## INTRODUCTION

Intracranial aneurysms occur rarely in children and adolescents. Although the literature on intracranial aneurysms in individuals younger than 18 years is limited and to some degree inconsistent, there seems to be consent in that pediatric aneurysms represent an independent condition different to their adult counterparts. Reports and institutional experiences with pediatric aneurysms are small case series ranging from three individuals [1] to 77 cases [2]. So far, until larger series applying a multi-center approach are performed and published, review of the literature is the best approach to understanding and recognizing this seldom condition. The present review focuses on intracranial pediatric aneurysms of non-traumatic origin as the latter actually are pseudoaneurysms caused by endothelial damage and thereby form a different pathofysiological entity. 

## ETIOLOGY 

In the adult population, certain risk factors are linked to the development of intracranial aneurysms, including smoking, arterial hypertension, age, gender and excessive chronic alcohol intake [3]. None of these are likely to contribute in the formation of aneurysms in children. Many pediatric intracranial aneurysms are related to arterial dissection. Cerebral arteriovenous malformations may carry aneurysms on feeding arteries. Approximately 5-10% of childhood cerebral aneurysms are related to head trauma [4].

Some comorbidities are related to a larger risk of aneurysm formation, amongst them reticular fiber deficiency [5], gene mutations interfering with the extracellular matrix [6] and connective tissue diseases including Marfan`s syndrome, coarctation of the aorta, Ehler-Danlos syndrome type IV and fibromuscular dysplasia [7]. Other associated medical conditions comprise polycystic kidney disease, tuberous sclerosis, sickle cell anemia, hereditary hemorrhagic telangiectesis (Osler-Weber-Rendu disease), Klippel-Trenaunay-Weber syndrome and alpha-1-antitrypsin deficiency. Underlying comorbidity is found in up to one third of the children with intracranial aneurysms [7-9]. 

Infectious etiology of an intracranial aneurysm (often denoted as “mycotic aneurysm”) is more common in children than in adults [4] and accounts for up to 15% of all pediatric aneurysms [4, 10, 11]. The most common infectious agens is bacterial, not fungal [4] and may be caused by bacterial endocarditis or syphilis. The most common causing organisms isolated are staphylococcus aureus and streptococcus viridans [12]. Children with immunodeficiency diseases like HIV may be especially prone to infectious aneurysms. [13-15]. By reviewing a total of 431 cases reported in the literature [7, 8, 11, 13, 16-29] we found 8,4% of the cases to be of infectious etiology.

## INCIDENCE

Pediatric intracranial aneurysms are acquired lesions as autopsy studies could not disclose aneurysms at birth [13]. The acquisition of an intracranial aneurysm during birth has been reported by Piatt and Clunie [30]. Aneurysms diagnosed in children younger than 1 year are extremely seldom, Buis *et al.* [9] found 131 cases in his review searching for publications from before 1966 until 2005. 

The incidence of aneurysmal hemorrhage was found to be 0.05-0.09 per 100000 person-years in children younger than 15 years, whereas it increases to 0.5 respectively, in those between 15 and 19 years [31]. Jordan *et al.* [31] numbers indicate a 35 times larger chance of aneurysmal hemorrhage in adults as compared to children. The fraction of pediatric aneurysms within the total volume of patients with intracranial aneurysms varies (Kakarla *et al.* 1% [16], Huang *et al*. [32] 1,4%, Sharma *et al.* 4% [33], Krishna *et al*. [17] 4,6%) with 0,95% in Fulkerson *et al*. `s [18] material and 6,8% in Lasjaunias *et al. *[13] series at the extremes. 

**Fig. (1):**
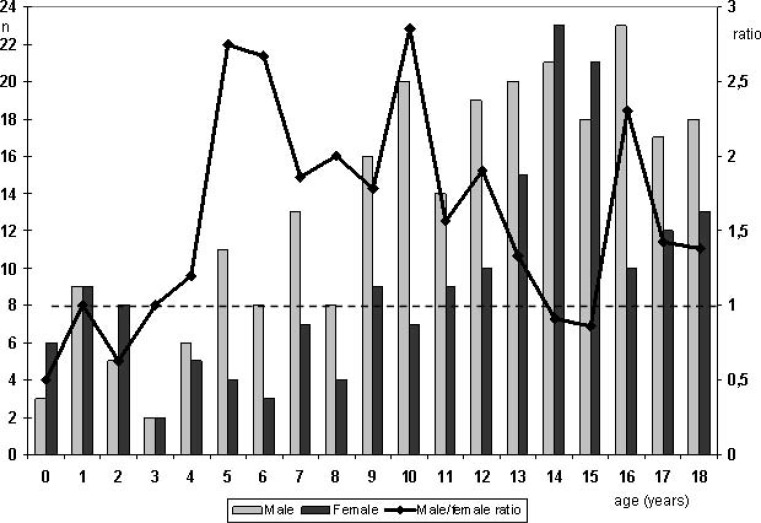
Distribution of age at diagnosis of the intracranial aneurysm in males (light grey columns) versus females (dark grey columns). Scale on the right shows the male/female ratio with values above “1” (dotted line) indicating male predominance. Data from [7, 8, 11, 13, 16-29].

Intracranial aneurysms in adults occur twice as often in females than in males [3]. This does not hold true in children. Lasjaunias *et al. *[13] observed a male to female ratio of 3:2, however, the ratio became 1:5 for children younger than 2 years. Krishna *et al. *[17]* r*eported a 1,75:1 male:female ratio, but they had no patients younger than 5 years. Another study from India, by Sharma *et al*. [33] found a 2,2:1 male:female ratio; however, most of their patients (82%) were older than 10 years, with only 4 children being younger than 5 years. Other authors reported a more even male/female distribution [7, 22]. Reviewing 431 cases reported in the literature [7, 8, 11, 13, 16-29] the male:female ratio is 1.42:1. The age distribution within sexes differs. Boys show a gradual increase in frequency with increasing age (Fig. **1**), whereas girls peak around menarche, resulting in a female predominance at ages 14 and 15 (Fig. **1**).

## CLINICAL PRESENTATION

Subarachnoid hemorrhage (SAH), is by far the most common presenting symptom of intracranial aneurysms [7, 8, 11, 16-18, 22, 28, 29, 33, 35-38]. In some reports, however, the rate of SAH in children was far lower, between 20-30% [2, 8, 39]. By reviewing 1165 published cases, 72% of those 0-18 years old presented with SAH (Table **1**), which is less than the 89% reported for the adult population [38]. 

The presentation with SAH has been claimed to be to some degree age-dependent with peaks at ages 2-5 and in adolescents older than 15 years [4]. Krings *et al.* [4] attributed this to the high incidence of dissecting aneurysms in those 2-5 years old and the prevalence of saccular “adult” aneurysms in those > 15 years. Fig. (**2**) illustrates the age distribution for aneurysmal hemorrhage, as accumulated from previous reports [7, 8, 11, 13, 16-29] with a higher frequency beyond the age of 10 but no clear peaks.

Re-bleeding before securing of the aneurysm seems to occur as often as in about half of the children [7, 11, 17, 40] and is thereby consistently more frequent than in adults where the incidence of re-bleeding varies between 16 and 29% [11, 41, 42]. 

Some authors found that children with aneurysmal intracranial hemorrhage more often present in a good clinical grade (Hunt and Hess grades 1-3, [43]) than adults [7, 8, 19, 20, 33, 26, 27, 44]. (Table **1**) shows that 68% of the children were in Hunt and Hess grades 1-3, a number not too different from the adult population. Fig. (**3**) summarizes the clinical grades from several reports providing adequate data [7, 8, 11, 13, 17-22, 24, 25, 27, 29], there was no statistical difference in clinical grade between girls and boys.

**Fig. (4):**
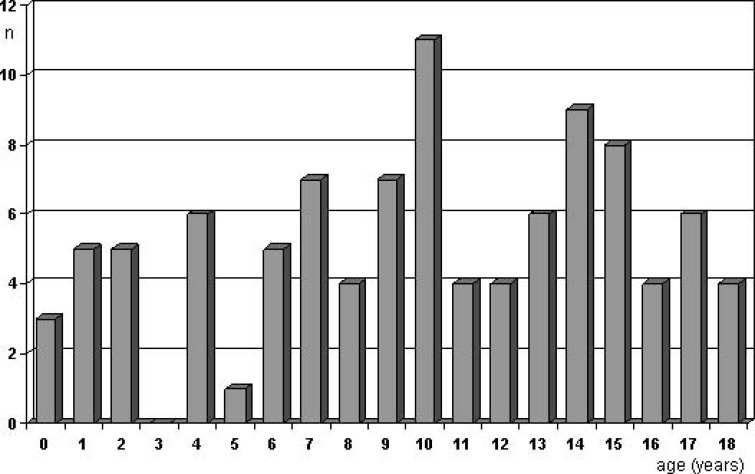
Age when the aneurysm became symptomatic (non-hemorrhagic, data collected from previous reports providing adequate information [7, 11, 13, 16, 18-21, 23-26]).

The lower incidence of hemorrhagic presentation in children is due to the high incidence of giant aneurysms that present with tumor effect rather than hemorrhage. Hence, mass effect from the aneurysm with various clinical neurological signs and symptoms is more common in children [4] but varies considerably between patient cohorts: 18,2% presented with symptoms referable to mass effect in the material of Sharma *et al.* [33], whereas the corresponding rate was as high as 46% in the patients of Kakarla *et al.* [16]. By reviewing 431 reported cases [7, 8, 11, 13, 16-29], we found that 17% of the children presented with neurological deficits and/or epilepsy, whereas in another 8% the aneurysm was found in the diagnostic work-up for chronic headaches. In 7% the aneurysm was an incidental finding. The rate of incidental diagnosis was as high as 35% in Kakarla *et al.* [16]. There was no clear age peak for when the aneurysm became symptomatic (Fig. **4**). The incidence of seizures in pediatric patients has been reported to be more than twice of that in adults (36% versus 17%, respectively [17]. Likewise, acute hydrocephalus was seen more often in children (36%) than in adults (25%) [17]. 

## ANEURYSM SITE

**Fig. (5):**
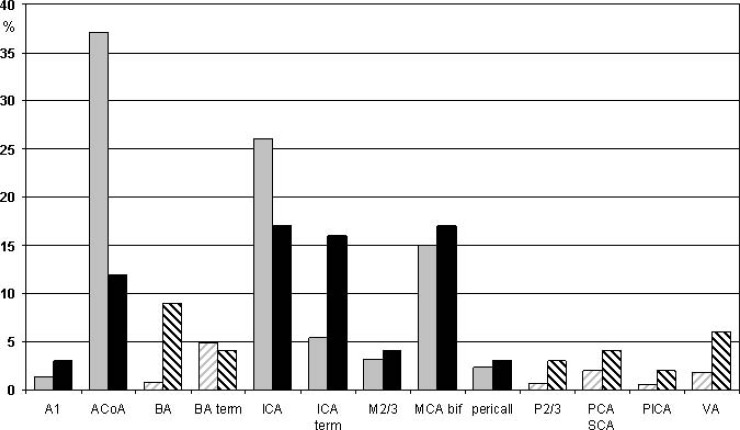
Localization of 671 pediatric (black columns) and 1021 adult (grey columns) intracranial aneurysms reported [7, 8, 11, 13, 16-29, 33, 36, 37, 39, 40, 44, 46]. Shaded columns indicate posterior circulation localizations. A1: anterior cerebral artery proximal to the anterior communicating artery (AcoA), BA: basilar artery (locations other than terminus), ICA: internal carotid artery (localizations other than terminus), M2 and M3: first and second branching of the middle cerebral artery (MCA), MCA bif: MCA bifurcation, P2/3: first and second branching of the posterior cerebral artery (PCA), PICA: posterior inferior cerebellar artery, SCA: superior cerebellar artery, VA: vertebral artery

There is consensus in that the anatomic sites of intracranial aneurysms in children differ from those in adults, although there are considerable variations between patient cohorts. Accounting for all internal carotid artery (ICA) locations (including the branching of the posterior communicating artery), the ICA is the most common site in children with 33%, therein a specific predominance of the ICA termination [4, 11, 27-29, 33, 34, 36, 45]. Krings *et al.* [4] found five times as many aneurysms on the ICA termination in children than in adults [4]. Reviewing 671 pediatric cases reported in the literature [7, 8, 11, 13, 16-29, 33, 36, 37, 39, 40, 44, 46], 16% of all pediatric aneurysms occur at the ICA termination alone (Fig. **5**), which compares to around only 5% in adults [38, 47]; i.e. the frequency of ICA termination (also denoted ICA bifurcation or ICA top) aneurysms is three times higher in children than in adults. 

In adults, the anterior communicating artery (ACoA) is the most common aneurysm site, accounting for 25-39% of all aneurysm locations [38, 47-49]. (Fig. **5**) shows that the ACoA in children is only the third most common aneurysm site with 12%. The second most common pediatric aneurysm site is the middle cerebral artery (MCA) bifurcation with 16% (Fig. **5**), which is quite similar to the frequency in adults. In infants <1 year of age, however, the MCA carried nearly 3 times as often an aneurysm as any other intracranial vessel [9]. Proust *et al*. [11] found MCA aneurysms to be the second most common site in children aged 7 to 16 years with 36% of their cases harboring MCA aneurysms; 50% of them were localized on the first MCA branch (M2 level).

Posterior circulation aneurysms are overrepresented in the pediatric population with a rate of 21%, which corresponds to almost three times the incidence found in adults [4, 38, 47] (Table **1**). (Fig. **5**) illustrates a frequency of 5% for vertebral artery aneurysms in children, whereas the corresponding number in adults is less than 2% [38, 47]. Likewise, aneurysms were found in 11,4% on the basilar artery in children (7,6% basilar artery + 3,8% basilar termination), but only in 4-8% in adults, respectively [38, 47]. 

Multiple aneurysms are found in approximately 14% of adult patients, with a range from 7-30% [38, 47]. This equals the findings of Lasjaunias *et al. *[13] with 15%, Sharma *et al.* [33] with 16%, and Kakarla *et al.* [16] with 31%. Others, however, reported a lower incidence of multiple aneurysms in children as compared to adults [7, 20, 50].

## ANEURYSM FEATURES

Giant aneurysms (≥ 25 mm) are found much more frequent in children than in adults. The numbers, however, vary widely from an incidence of zero to 68% [7, 8, 11, 16, 20, 23, 28, 33, 40, 44]. Krishna *et al. *[17] reported the incidence of giant aneurysms in children to be 13,6%, whereas the corresponding number in adults was 6,5%. Proust *et al.* [11], on the other hand, found a frequency of giant aneurysms around 14% in both pediatric and adult patients. In infants <1 year, the mean size of aneurysms was 18mm, with 30/131 being giant [9]. (Table **1**) shows that the frequency of giant aneurysms in children and adolescents is reported to be 19%. Pediatric giant aneurysms seem to favor the vertebrobasilar arteries with reported incidences in that specific location ranging from 8-100% [10, 11, 19, 20, 23, 26, 36, 37, 44, 45, 51, 52]. 

Dissecting and fusiform aneurysms are relatively common in children in contrast to adults who typically carry saccular aneurysms. In the material of Lasjaunias *et al. *[13] the fraction of dissecting aneurysms in children was as high as 45%. Krings *et al. *[4] stated that the incidence of dissecting aneurysms is four times higher in children than in adults. Fusiform or dolichoectatic aneurysms accounted for 51% of the aneurysms in the pediatric patients of Sanais *et al.* [8]. Non-traumatic dissecting aneurysms are most commonly found at the level of the supraclinoid ICA, the MCA, and in the posterior circulation, preferably the P1and P2 segments of the posterior cerebral artery [53]. MCA aneurysms in children tend to be large and/or ectatic, often including M2 branches in the pathological vessel section [18].

The rate of infectious aneurysms (often referred to as “mycotic” aneurysms) is higher in children than adults (8% versus 5%) [9, 17, 38]. As this type of aneurysm usually arises from the arterial adventitia, they emerge often as fusiform or «false» aneurysms [4]. Infectious aneurysms are often situated at distal arterial branches and may be multiple. 70% of the infectious aneurysms present with hemorrhage. 

## TREATMENT

The treatment of pediatric patients with intracranial saccular aneurysms seems to follow the institutional protocols developed for adult aneurysm patients [11, 33, 54]. Accordingly, treatment often is dependent on the traditions of the neurosurgical center and ranges from surgically clipping all patients [7, 17, 36] to endovascular treatment in all [23] or the majority of the patients [13]. 

The long life expectancy of children challenges in particular the durability of treatment. In this respect, surgical treatment may be superior to endovascular approaches [16]. Sanai *et al.* [8] found that children treated endovascularly were four times as often in need to further treatment as compared to those that were surgically treated. Stiefel *et al.* [22] preferred to clip pediatric aneurysms (except those on the basilar artery) because they were not convinced of the durability of endovascular treatment. Likewise Sanai *et al.* [8] built a strong case for surgical treatment as they observed a higher recurrence rate in endovascularly treated aneurysms along with a higher rate of de novo aneurysm formation in children. They attributed the latter to catheter manipulation causing small arterial wall defects as the potential source of aneurysm formation [8]. 

The higher fraction of complex aneurysms in children requires more often advanced treatment beyond standard aneurysm clipping or coiling, including intracranial and extracranial-intracranial bypass procedures, hypothermic arrest and aneurysm resection [8, 11, 16, 19, 44]. Pediatric patients represented 10.2% of all cardiac standstill procedures in the material of Karkarla *et al.* [16] due to the large size and posterior fossa localization of many of the aneurysms. By reviewing 431 published pediatric cases, there emerges a clear preference for surgical treatment accounting for 55% of all cases versus 12% that were treated endovascularly [7, 8, 11, 13, 16-29]. Sixteen percent were treated with therapeutic parent artery occlusion, 13% were managed conservatively, whereas 4% had antibacterial treatment only [7, 8, 11, 13, 16-29]. On the other hand, it is reasonable to assume that the long-term effect of endovascular treatment of pediatric patients is not yet established due to the small number of patients treated, the relatively short time of follow-up, and the enormous evolution of endovascular techniques [55]. 

Therapeutic artery occlusion is a quite common treatment option in pediatric aneurysm patients, with numbers as high as 64,7% for parent artery sacrifice [13, 23] and 11,8% proximal artery occlusion with flow reversal in the artery carrying the aneurysm [13]. This treatment option was chosen in 16% of reported cases [7, 8, 11, 13, 16-29]. The tolerance to therapeutic vessel occlusion is reported to be better in children than in adults [18, 26, 28]. 

Lasjaunias *et al. *[13] and Liang *et al.* [20] reported on a high fraction of pediatric patients in which the aneurysm had thrombosed spontaneously, which is a rather uncommon event in the adult population. In dissecting aneurysms, mural hematoma seems to be the most important promoting factor for spontaneous thrombosis and healing of the aneurysm [21].

A very important peculiarity that needs to be mentioned is the tendency to delayed treatment in children [11, 17]. Krishna *et al*. [17] observed an interval of more than 14 days from ictus to surgery in 41% of their pediatric patients, compared to 27% of the adult patients. Delayed diagnosis of aneurysmal SAH increases the chance of re-hemorrhage from the unsecured aneurysm, with all its possible deleterious effects, thereby seriously affecting outcome.

## OUTCOME

Many authors report a more favorable outcome in the pediatric patient cohort than in adults. There is, however, considerable variation between reports. Lv *et al.* [23] obtained a good outcome in 96% of the pediatric patients by predominantly securing the aneurysms with parent artery occlusion (including 3 cases of MCA occlusion), however 14 of their 25 patients had not hemorrhaged and the clinical state of those with ruptured aneurysms is not given. It is worth noting that results in the adult subgroup selected for treatment with parent artery occlusion are equally good with zero one year mortality [56, 57]. In the material of Krishna *et al. *[17], 82% of the children had a favorable outcome versus 59% of the adults. Comparing outcome in children and adults one needs to consider that a large portion of the children had non-ruptured aneurysms where outcome can be anticipated to be different from that in subjects that have suffered hemorrhage. 

Approximately 90% of the children that had their aneurysm treated were in Glasgow outcome scale (GOS, [58]) 5 or 4 (i.e. excellent or good result) in the reports by Sharma *et al.* [33] and Liang *et al.* [20]. Outcome in infants younger than one year was similar or slightly better than in adults with 50% in GOS 5 [9]. In parallel to the treatment results in adults, the outcome in children has improved over the last decades [35] due to a shift to early securing of the aneurysm, the development of microsurgical techniques and endovascular treatment options, along with improvements of post-operative care. 

Overall, a good outcome, defined as GOS 5 or 4 is reported in 68% of children with intracranial aneurysms (Table **1**), this number, however, includes reports merely considering outcome of survivors. The true fraction of those with good outcome is hence lower. 

**Fig. (6):**
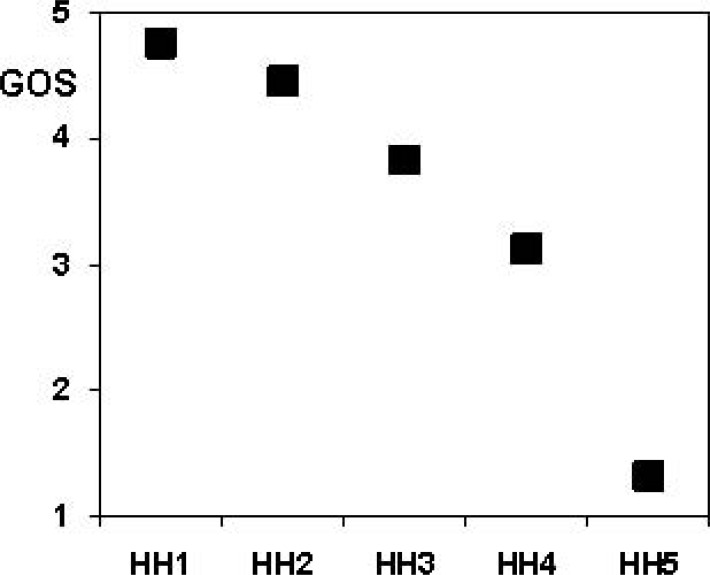
Most children that were in good clinical grade (Hunt and Hess [43] grades 1-3) had an excellent (GOS5) or good (GOS4) outcome, whereas many of the children presenting in a poor clinical grade (Hunt and Hess grades 4 and 5) died (GOS1) or survived to a dependent life. GOS: Glasgow outcome scale [58]. HH: Hunt and Hess grade.

In case of ruptured aneurysms, (Table **2**) the clinical grade immediately following the SAH is a strong predictor of outcome ((Fig. **6**), p<0.0001 Kruskal-Wallis test).

None of the children in Hunt and Hess grade 5 (i.e. those who are immediately comatose from their hemorrhage) survived to a good clinical outcome and mortality in that subgroup was 82% [7, 8, 11, 13, 16-29]. Mortality numbers in children versus adults seem to be similar [7, 11, 16, 17, 22]. In infants <1 year mortality was 28% [9]. Approximately 12% of adults with aneurysmal SAH succumb to their bleed before they can reach medical attention [32, 59]. The corresponding number in children is unknown.

Cerebral vasospasm represents a major risk factor for poor outcome or death following SAH. Angiographic vasospasm was observed equally in children and adults, however, clinical vasospasm is more infrequent in children [11, 17, 60]. Krishna *et al. *[17] found clinical vasospasm in 9,5% of their pediatric patients versus 28,5% in adults, respectively. Proust *et al. *[11] described six children with angiographic severe vasospasm that all were asymptomatic. Lasjaunias *et al.* [13] observed that the tolerance to cerebral vasospasm was good in children, even after treatment with therapeutic parent artery occlussion which poses an extra challenge with regard to developing cerebral ischemia. In the material of Sharma *et al. *[33], 10/43 children were diagnosed with vasospasm and 20% of them developed manifest cerebral infarction. Their findings concur well with that of Stiefel *et al.* [22]; who observed a 17% frequency of stroke due to vasospasm, wherein all occurred in children younger than 2 years.

The long-term prognosis of children with intracranial aneurysms is influenced by a higher risk to develop de novo aneurysms than adults [14]. Hetts *et al. *[14] found that 8,4% of their pediatric patients developed new or enlarging aneurysms within an average of less than five years of the initial diagnosis. This compares to a corresponding 1,5% rate reported in adults [61]. Kakarla *et al. *[16] found an annual recurrence rate of 2,6% with an annual rate of de novo formation or aneurysm growth of 7,8% in children.

## DISCUSSION

Pediatric intracranial aneurysms are very rare, with approximately 1200 cases reported between 1939 and 2011 worldwide. No real large series or multicenter studies so far exist and reports on pediatric intracranial aneurysms reflect institutional experiences acquired over a large time-span or are published as case reports. Consequently, the results from the various studies are inconsistent and partly divergent. One could also presume that it is not only the limited number of patients, but also differences in institutional and regional referral practices that contribute to the diversity of findings. Furthermore, treatment strategies and the available treatment armamentarium has changed dramatically over the last decades and reports from the 60ies should not be compared with reports from the current century.

There is consensus, though, in that pediatric aneurysms represent a pathophysiologic entity different from their adult counterparts - they are not simply "standard" saccular aneurysms occurring in very young humans. This also means that management strategies acquired for adults cannot be fully transferred onto the pediatric patient population. First, pediatric aneurysms most often are complex and hence require more challenging treatment strategies like intracranial bypass procedures or therapeutic parent artery occlusion. Approximately one third of the aneurysms are giant, exerting significant tumor effect to delicate brain structures, predominantly in the posterior fossa. Second, regarding the long life-expectancy of pediatric patients, the durability of treatment is more crucial than in the typical adult aneurysm patient that is in its 5th decade of life when becoming symptomatic. Endovascular treatment with coils or coiling through a stent may not accomplish life-long occlusion of the aneurysm. Furthermore, endovascular procedures are difficult to perform in the youngest children and many centers may not have sufficient experience to treat the child themselves. Who should treat children with intracranial aneurysms? This question is difficult to answer in general terms and would have to be decided individually, based and the specific nature of the aneurysm and the availability of treatment options at the hospital the child is admitted to. Many institutions do not have a 24 hour availability of both cerebrovascular surgeons and endovascular therapists. In that regard, one also has to keep in mind that many pediatric aneurysms are complex and pose special requirements to the skills of the therapist. For sure, the pediatric aneurysm patient requires a broader multidisciplinary cooperation involving vascular neurosurgeons, neurointerventionalists, pediatricians, pediatric ICU staff, psychologist and a rehabilitation team with experience in treating pediatric stroke.

There is another challenge to pediatric aneurysm patients too: this condition is so rare that aneurysmal SAH would not necessarily be included in the diagnostic armamentarium when a child presents with headache. This can lead to both patient and doctor related delay in diagnosis and treatment. Accordingly, there is a higher rate of re-bleeding of ruptured aneurysms in children than in adults [7, 11, 17, 40] which may effect the outcome dramatically to the worse. The reported numbers do not include the children that never made it to a hospital after their bleed or re-bleed. In the adult population an immediate death toll of approximately 12% of all aneurysmal SAH is anticipated [32], one could assume this rate to be similar or higher in the pediatric population. To prevent re-bleeding, children should be diagnosed as early as possible, i.e. pediatricians should keep the possibility of aneurysmal hemorrhage in mind when a child presents with sudden headache. In the presence of nuchal rigidity, a lumbar puncture, CT scan, or MRI would be performed, usually leading to the correct diagnosis. With that clinical presentation, if meningitis is suspected and a lumbar puncture is performed one should also include analysis for xanthochromia. There would be a larger problem if the child is in a relatively good clinical condition apart from the headache, as routinely screening with CT or MRI would not necessarily be justified. Less than 3% of cases with adult sudden headache are due to aneurysm bleeds [62], and the chance of finding intracranial hemorrhage in a child, respectively, would be even less. Individuals younger than 18 years seeking medical attention for headache account for 0,8% of all emergency department visits in the United States [63], with a pathological finding in less than 2% of them [63]. On the other hand, as long as the diagnosis of a ruptured aneurysm is not established, the danger of re-bleed is eminent. Re-bleeding in adults is identified as a strong predictor of poor outcome and death.

The mortality in children and adults is reported to be similar. Likewise, studies that included children with a clinical presentation comparable to the adult aneurysm cohort found similar outcomes [11, 22]. One could hence suspect the notion of favorable outcome in children with intracranial aneurysms to be a myth. Another predictor of poor outcome is the occurrence of severe vasospasm. There are several reports on the good tolerance of cerebral vasospasm in children, which should give them an advantage in terms of outcome compared to adults. Moreover, patients with ruptured intracranial aneurysms have a higher mortality and poorer outcome than those without intracranial hemorrhage unless the aneurysm is giant. Giant aneurysms in adults, even if unruptured, have a poor prognosis and a mortality rate of up to 66%, with those in the posterior circulation having the poorest odds of all [56, 64]. A possible explanation for children not having a lower mortality and morbidity could be the high percentage of giant aneurysms in the posterior circulation in children. Another pitfall in establishing outcome in children with cerebral aneurysms are differences in outcome measures between various reports. In many studies, outcome has been considered as "good" or "excellent" without using any standardized mode of measurement or rigorous criteria for assessment. None of the reports included neuropsychological investigations. In adults, even patients scored as excellent or good outcome (GOS 5 or 4) by a neurosurgeon may display significant impairments on neuropsychological tests and in Quality of life questionnaires [65]. In children one could assume similar effects on brain function or even worse impact in very young children whose brain is still developing and thereby could be more susceptible to the consequences of hematoma, treatment trauma and secondary ischemic damage. Outcome will also have to be dichotomized into early outcome, i.e. up to one year past diagnosis versus the long-term outcome. In many reports data for the long-term results in pediatric aneurysms were, however, limited. Long-term outcome will be strongly influenced by the high rate of aneurysm recurrence and de novo aneurysm formation in children. 

Cerebral aneurysms in children should be considered an aggressive, chronic condition. Some of the de novo aneurysms formed early after the initial debut of disease, many within 3 years [16]. The high rate and pace of de novo aneurysm formation in children should naturally affect the follow-up of the pediatric population. So far, the literature, however, indicates that most children are lost to follow-up after relatively short time without any obvious enrollment in a screening sequence. We agree with Kakarla *et al. *[16] that life-long follow-up with MRI- or CT angiography is mandatory and would suggest imaging for screening purpose at three year intervals.

In conclusion, there are certain peculiarities about pediatric intracranial aneurysms that make them differ from their adult counterparts. More consensus on the nature of this rare condition will, however, not evolve until data are collected in national databases and united in a large international multicenter trial. Even with such an approach, considerable time and efforts will be consumed until sufficient amounts of data are available.

## Figures and Tables

**Fig. (2):**
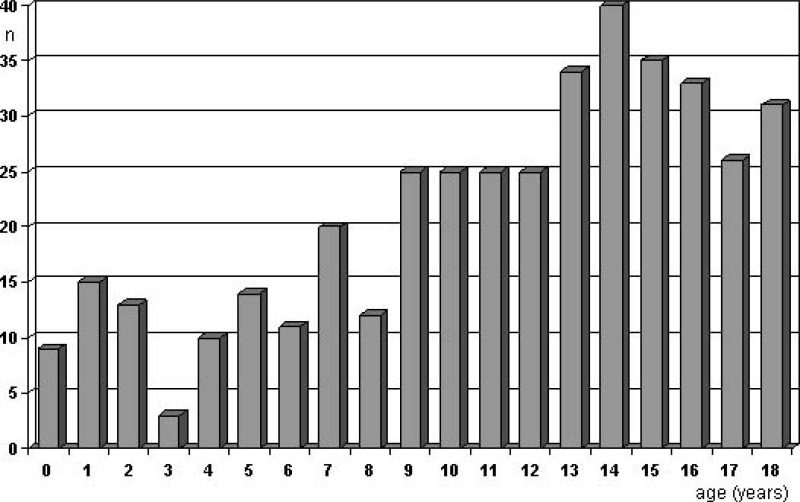
Age at aneurysm rupture (data collected from previous reports providing adequate information [7, 8, 11, 13, 16-29]).

**Fig. (3):**
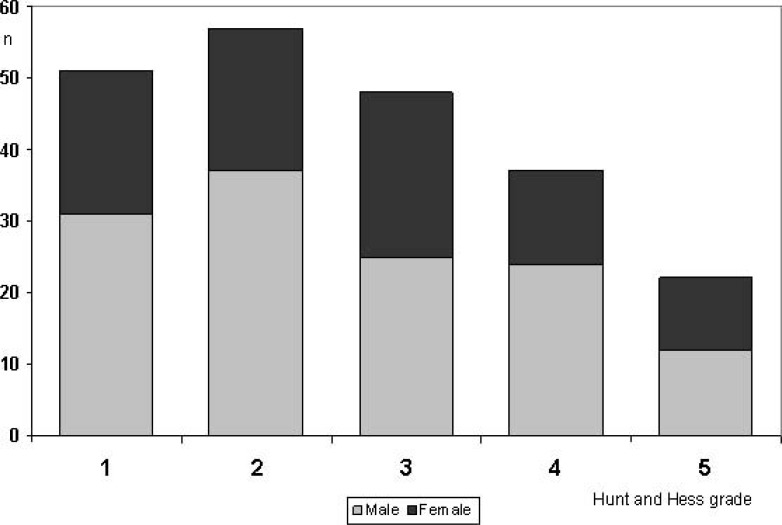
Clinical grade according to Hunt and Hess [43] in males (light grey) and females (dark grey) with ruptured intracranial aneurysms (data collected from previous reports providing adequate information [7, 8, 11, 13, 17-22, 24, 25, 27, 29]).

**Table 1.** Summary of Publications on Pediatric Intracranial Aneurysms

Year	Authors	Cases n=	Males (%)	SAH (%)	Good grade (%)	Giant aneurysm (%)	Posterior Circu-lation (%)	Good outcome (%)	Mortality (%)
1939-2009	Accumulated data From the review of Jian *et al.* [35] Range given in ( )	965 (3-77)	60 (33-92)	74 (33-100)	51 (0-96)	21 (0-54)	19 (0-46)	62 (13-95)	24 (0-100)
2007	Sharma *et a.l* [33]	55	69	78	86	19	12	90	9
2008	Songsaeng *et al.* [21]	8	63	25	63	25	50	63	0
2008	Stiefel *et al.* [22]	12	33	100	75	8	33	75	17
2009	Liang *et al.* [20]	24	58	46	88	32	36	92	4
2009	Lv *et al.* [23]	25	80	44	Not given	68	60	96	4
2010	Kakarla *et al.* [16]	48	58	35	83*	23	24	92	3
2011	Fulkerson *et al.* [18]	28	43	68	71	7	21	92	7
Total		1165	65	72	68	19	21	68	19

* : based on GCS scores at admission

**Table 2.** Outcome Accumulated from Reports [16, 18, 20-23, 33, 35] Providing Adequate Data. GOS: Glasgow Outcome Scale [58]

	Good result - independent		Poor result - dependent		dead
	GOS 5	GOS 4	GOS 3	GOS 2	GOS 1
Unruptured aneurysms	66%	21%	7%	1%	5%
Hunt and Hess grade 1	85%	11%	2%	0	2%
Hunt and Hess grade 2	68%	22%	2%	2%	6%
Hunt and Hess grade 3	44%	30%	10%	2%	14%
Hunt and Hess grade 4	12%	30%	28%	9%	21%
Hunt and Hess grade 5	0	0	13%	5%	82%

## ABBREVIATIONS

ACoA: = anterior communicating artery
A1: = anterior cerebral artery proximal to the AcoA
BA: = basilar artery
CT: = computed tomography
GOS: = Glasgow outcome score
HIV: = human immunedeficiency virus
ICA: = internal carotid artery
MCA: = middle cerebral artery
M2 and M3: = first and second branching of the MCA
MRI: = magnetic resonance imaging
SAH: = subarachnoid hemorrhage
PCA: = posterior cerebral artery
Pericall: = pericallosal artery
P2 and P3: = first and second branching of the PCA
PICA: = posterior inferior cerebellar artery
SCA: = superior cerebellar artery
VA: = vertebral artery
